# Discovery of a New Genetic Variant of Methionine Aminopeptidase from *Streptococci* with Possible Post-Translational Modifications: Biochemical and Structural Characterization

**DOI:** 10.1371/journal.pone.0075207

**Published:** 2013-10-04

**Authors:** Tarun Arya, Chandan Kishor, Venkateshwarlu Saddanapu, Ravikumar Reddi, Anthony Addlagatta

**Affiliations:** Centre for Chemical Biology, CSIR-Indian Institute of Chemical Technology, Hyderabad, Andhra Pradesh, India; University of Kansas Medical Center, United States of America

## Abstract

Protein N-terminal methionine excision is an essential co-translational process that occurs in the cytoplasm of all organisms. About 60-70% of the newly synthesized proteins undergo this modification. Enzyme responsible for the removal of initiator methionine is methionine aminopeptidase (MetAP), which is a dinuclear metalloprotease. This protein is conserved through all forms of life from bacteria to human except viruses. MetAP is classified into two isoforms, Type I and II. Removal of the *map* gene or chemical inhibition is lethal to bacteria and to human cell lines, suggesting that MetAP could be a good drug target. In the present study we describe the discovery of a new genetic variant of the Type I MetAP that is present predominantly in the streptococci bacteria. There are two inserts (insert one: 27 amino acids and insert two: four residues) within the catalytic domain. Possible glycosylation and phosphorylation posttranslational modification sites are identified in the ‘insert one’. Biochemical characterization suggests that this enzyme behaves similar to other MetAPs in terms of substrate specificity. Crystal structure Type Ia MetAP from *Streptococcus pneumoniae* (*Sp*MetAP1a) revealed that it contains two molecules in the asymmetric unit and well ordered inserts with structural features that corroborate the possible posttranslational modification. Both the new inserts found in the *Sp*MetAP1a structurally align with the P-X-X-P motif found in the *M. tuberculosis* and human Type I MetAPs as well as the 60 amino acid insert in the human Type II enzyme suggesting possible common function. In addition, one of the β-hairpins within in the catalytic domain undergoes a flip placing a residue which is essential for enzyme activity away from the active site and the β-hairpin loop of this secondary structure in the active site obstructing substrate binding. This is the first example of a MetAP crystallizing in the inactive form.

## Introduction

**Figure 1:**
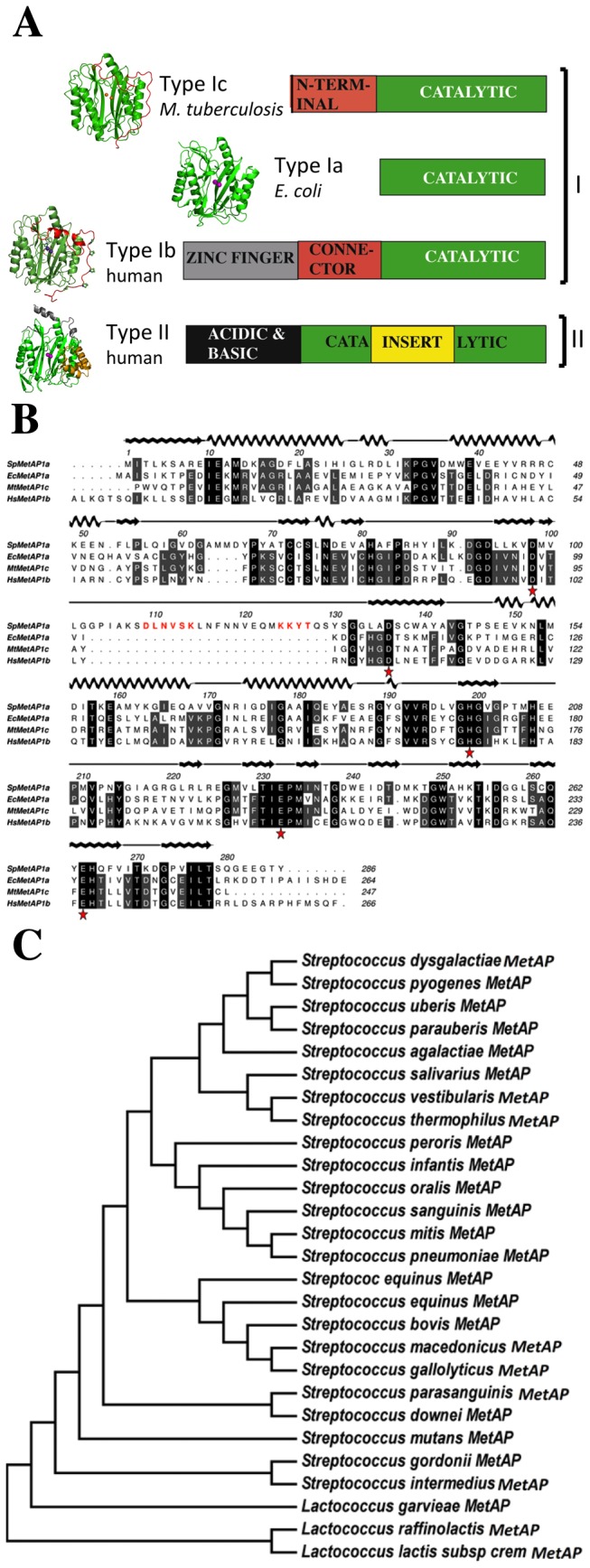
a) Representation of domain structure of sub-classes of MetAP based on the crystal structures. Common catalytic regions are shown in green. Type Ia has the basic structure required for catalysis. Type Ib and Type Ic have extra regions on the amino terminus. Type II MetAP has a 60 amino acid insert in addition to N-terminal extension (yellow). On the left of each bar is the representative crystal structure in cartoon diagram. Pink spheres in the middle indicate the two metal ions in the active site. b) Sequence alignment of Type I MetAPs from *S. pneumoniae* (Type Ia), *E. coli* (Type Ia), *M. tuberculosis* (Type Ic) *and* *human* (Type Ib). Numbering and the secondary structure representation on the top of the alignment are based on the *Sp*MetAP1a crystal structure. Metal binding conserved residues are marked as asterisk. Two inserts are identified^63^; insert and ^103^insert. These inserts are predominantly present in the streptococcus bacterial Type Ia MetAP^109^. DLNVSK and ^124^KKYT are two sequences marked in red are predicted to have respectively glycosylation and phosphorylation sites. c) Phylogenetic tree of 24 Type I MetAPs from streptococcal and lactococcal bacteria that contain the two inserts observed in the Figure 1b. Note that each of these species forms separate clusters.

Almost all proteins in living cells are synthesized by the ribosome. The first amino acid during this synthesis is a formyl methionine in eubacteria and methionine in eukaryotes. In about 60-70% of newly synthesized proteins, the initiator methionine is removed by a metalloenzyme called methionine aminopeptidase (MetAPs) [1]. In bacteria, excision of methionine is preceded by the removal of formyl group by a deformylase. Disruption of the MetAP function is detrimental [2,3]. Except for actinobacteria, all other bacterial species encode for a single *map* gene classified as MetAP1a. Actinomyces carry additional gene for MetAP classified as MetAP1c (Figure 1a) [4]. The difference between MetAP1a and MetAP1c is the presence of an additional 40 residue amino-terminal extension that carries consensus SH3 binding P-X-X-P motif suggesting its capability to interact with other proteins [4]. Except for these polyproline regions no other structural motifs have been identified in bacterial MetAPs that may participate in protein-protein or protein-nucleotide interactions. All eukaryotic proteins have been demonstrated to carry additional protein sequences apart from the catalytic region that enables them to bind to the ribosome and co-translationally remove the initiator methionine (Figure 1a) [5].

There has been a great interest in understanding the protein synthesis and co-translational peptide modification at the ribosome exit-tunnel [6]. Recent structure of ribosome in complex with the deformylase provided the glimpse of N-terminus of the peptide modification at the ribosome exit tunnel in bacteria [7]. Recently, it was shown through structure and biochemistry, that bacterial MetAP associate with ribosome for co-translational removal of the initiator methionine in the same place where the deformylase would associate [8].

We have long-term interest in understanding different genetic variants with new functional motifs in MetAPs that can link their ability to interact with other macromolecules, specifically to the ribosome [4,5]. Here, we report the discovery of a novel MetAP1a found predominantly in the streptococci bacteria with two new structural motifs one of which may undergo posttranslational modification of glycosylation and phosphorylation. We demonstrate that despite of these extra-motifs near the active, enzyme displays strict specificity to only methionine like other MetAPs. In addition, we report that this enzyme crystallizes in the inactive conformation, which is a first observation among more than 55 crystal structures reported so far.

## Results

### Bioinformatic analysis of SpMetAP1a

In order to identify new genetic variants of Type Ia MetAP, exhaustive search of the genomic databases using various bioinformatics tools including BLAST and multiple sequence alignment lead to the discovery of insert of about 27 amino acids within the catalytic domain (Figure 1b) [9,10]. This insert is present specifically in all streptococci bacterial MetAP (^102^GGPIAKSDLNVSKLNFNNVQMKKYTQSYSG in *Streptococcus pneumoniae* (*Sp*MetAP1a) apart from streptococci, three lactobacillus (^103^GLVEDGSVDVSKLDFDDAESMVKYQE in *Lactococcus lactis*) strains Figure 1b and Figure 1c). Note that traditionally, streptococci and lactobacillus were part of the same genus until recently [11]. Search of Uniprot database confirms that these microbes contain a single gene of *map* unlike the actinomyces family of bacteria [4,12]. In the rest of this paper, we will discuss about the MetAP from streptococcal bacteria only. BLAST search and multiple sequence alignment of the 27 amino acid insert (based on top 100 hits) revealed a pattern of conservation^109^d/e-V-s/t-k/r-L-d/e-F-n/q-(X)_5_-K, where the capital letter indicates the absolute conservation of a particular residue and two lower case letters indicate the variation of these residues at a particular position and X is any amino acid. In addition to this large insert, a four amino acid small insert is also noticed at^63^AMMD.

Type Ia MetAP is the isoform with minimum sequence and structure required for the catalysis and is present in all prokaryotes. Both Type Ib and Type II MetAPs present in the eukaryotes have extra-regions in the sequence, which are predicted to interact with other macromolecules to give extra functionalities to these enzymes (Figure 1a). It is for the first time that Type Ia MetAP is discovered to have an extra-sequence within the catalytic domain (^103^Gly–Ser^129^ in *Sp*MetAP1a) (Figure 1b). Analysis of this region using various post-translational modification (PTM) databases suggests the possibility of at least two sequon (^109^DLNVSK^114^ and^124^KKYT^127^) that may undergo post-translational modification. The consensus sequences, N-X-S/T (where X is any amino acid other than proline) in eukaryotes and D/E-X-N-X-S/T in prokaryotes are very well known glycosylation motifs where the asparagine gets glycosylated. The KKYT is a tyrosine kinase phosphorylation motif [13].

### Biochemical characterization of *SpMetAP1a*

**Figure 2:**
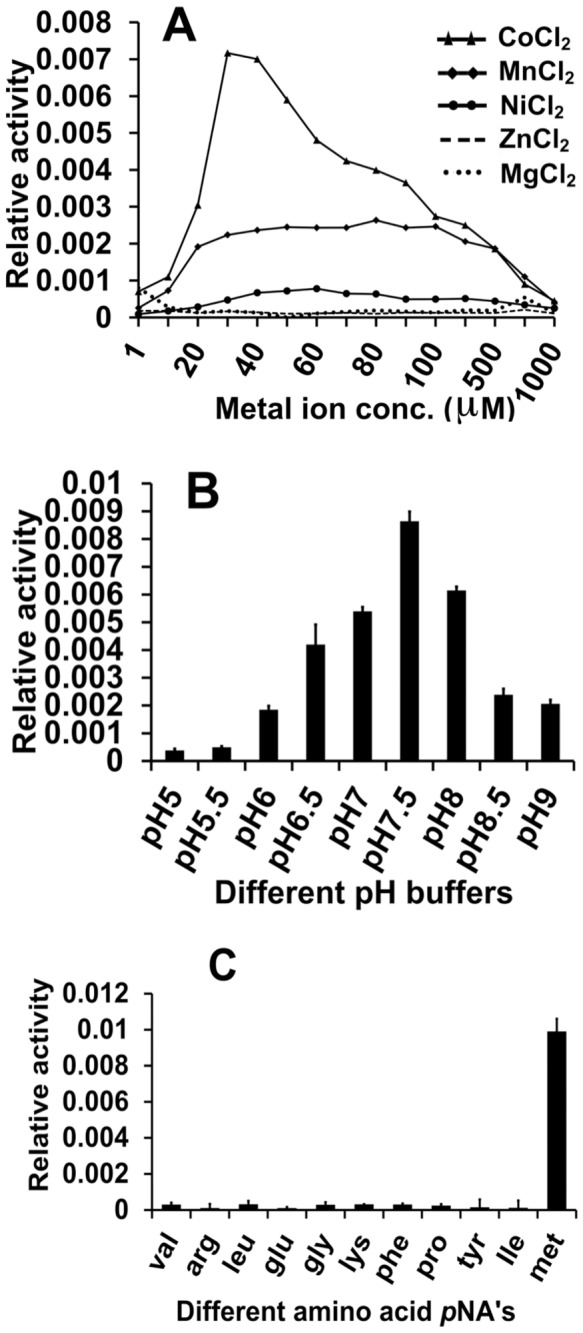
Biochemical characterization of *Sp*MetAP1a. a) Enzyme binding curve of the Met-pNA at varied concentrations is depicted with the fit value of 0.97924. b) Metal dependency on the activity of the enzyme is tested using five different metal salts ranging from 1-1000 µM. Cobalt is identified as the best co-factor followed by manganese. In the presence of nickel, negligible activity was observed. Zinc and magnesium did not activate the enzyme. c) Activity dependency of the *Sp*MetAP1a on pH change. Maximum activity was observed at pH 7.5. d) Substrate specificity of the *Sp*MetAP1a. Among the eleven amino acid-*p*NA substrates tested, only methionine was hydrolyzed.

MetAPs have strict substrate specificity towards peptides with methionine on the amino-terminus. These are metalloenzymes with preference for first row transition metal ions. To understand the substrate specificity, metal ion preference and catalytic efficiency, we have carried out biochemical assays on the purified recombinant *Sp*MetAP1a (Figure 2, Table 1). Best enzyme activity was detected in the presence of cobalt followed by manganese. With cobalt maximum activity was observed in the presence of two-equivalents of metal ion with respect to enzyme concentration. Enzyme displayed optimum activity at pH = 7.5 in 25 mM HEPES buffer. Eleven para-nitroanilide (*p*NA) derivatives of different amino acids (Val, Arg, Leu, Glu, Gly, Lys, Phe, Pro, Tyr, Ile and Met) that include small, hydrophobic, polar and charged side chains were used to explore the substrate specificity. As expected, the enzyme hydrolyzed only methionine-*p*NA. This suggests that the two insert regions do not affect the specificity of the enzyme.

**Table 1:** Kinetic data for *Sp*MetAP1a.

*K*_*m*_ (µM)	513.4 ±1.28
*K*_*cat*_ (min^-1^)	0.331 ± 0.003
Kcat/Km (nM^-1^ min^-1^)	0.647

### Crystal Structure determination of SpMetAP1a

#### Overall structure

**Figure 3:**
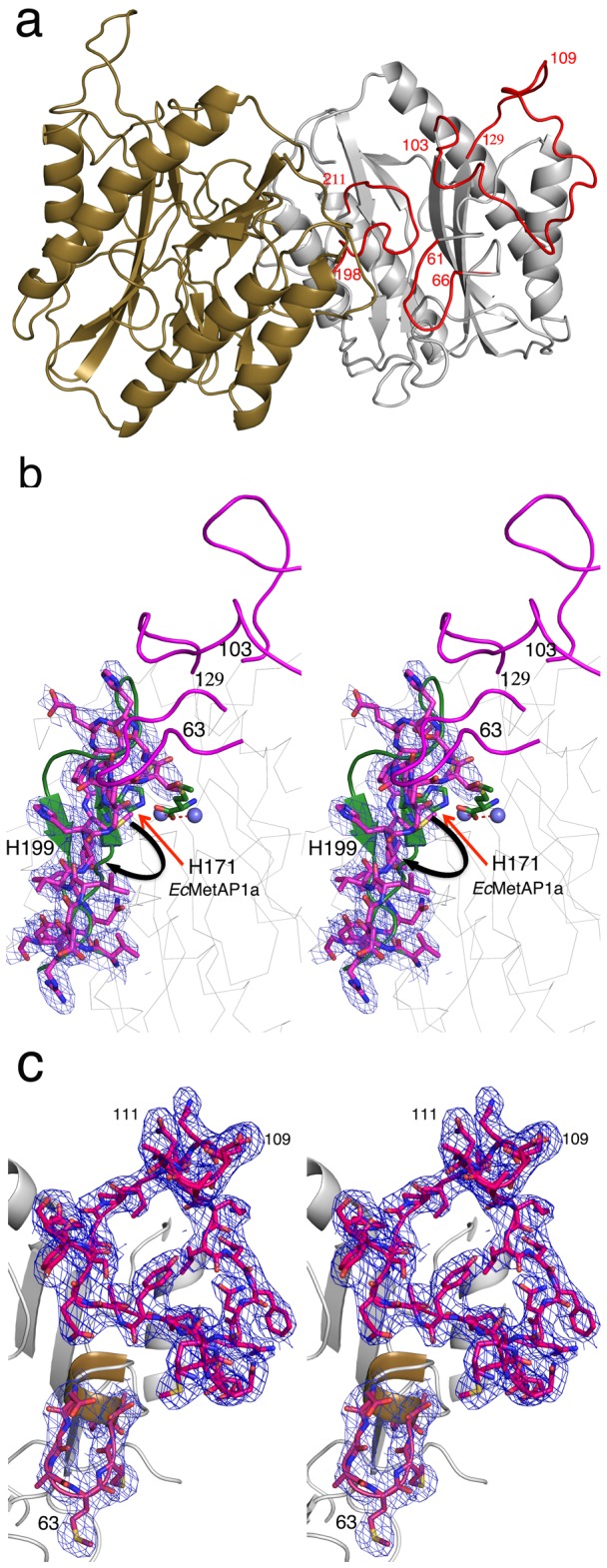
Crystal structure of *Sp*MetAP1a. a) Cartoon representation of two molecules (brown and gray) in the asymmetric unit. Two inserts^63^, insert and ^103^insert are shown in red in one of the molecules. The overall pita-bread fold is conserved like in other known MetAPs. b) Stereo diagram of the two inserts on the top and the^198-211^β-hairpin (equivalent region in *Ec*MetAP1a is 170-183 is shown in brown) undergoes a conformational change and occupies the active site where the substrate methionine side chain usually binds while the metal binding H199 moves away from the active site. The 2*F*o-*F*c electron density is shown at 1.2 σ. Two metal ions (blue spheres) and the product methionine (brown sticks) are shown from the *Ec*MetAP1a crystal structure. The black arrow shows the direction of the hairpin flip. The red arrow points to the histidine that is essential for the activity but flips away in the *Sp*MetAP1a structure (H199). c) Electron density cover (2*F*o-*F*c) of^63^insert and ^103^insert at 1.0 σ. The tight helix turn in *Ec*MetAP1a (brown helix) extends into a longer loop in the^63^insert. Reverse β-turn is represented near 109-111 residues.

To further understand the relationship of the two inserts with respect to catalytic domain, crystal structure of the *Sp*MetAP1a is determined at 3.2 Å in the *P*6_5_ space group with two molecules in the asymmetric unit (Figure 3a, Table 2). The overall structure is similar to that of the *E. coli* MetAP1a (*Ec*MetAP1a) adopting the pita-bread fold. Since the crystals were grown in the absence of metal salts, the active site is devoid of any metal ions. Attempts to crystallize the protein in the presence of CoCl_2_ did not yield diffraction quality crystals. The metal binding residues are well ordered and interact with each other as was noticed in the human MetAP1b apo structure [5]. Density is clearly visible for all N-terminal and C-terminal residues of the protein in both the molecules in the asymmetric unit. Root mean square deviation (rmsd) between A and B molecules in the asymmetric unit is 0.75 Å. Similarly, the rmsd with *Ec*MetAP1a, *Enterococcus feacalis* MetAP1a (*Ef*MetAP1a), *Mycobacterium tuberculosis* MetAP (*Mt*MetAP1c) and *Sp*MetAP1a (molecule A in the asymmetric unit) is 1.45 Å, 1.52 Å and 1.58 Å, respectively.

**Table 2:** 

**Cell parameters**	
Space group	*P*6_5_
*a,b* (Å)	109.69
*c* (Å)	164.42
**Data collection**	
Resolution range (Å) (Highest res. shell)	23.7 − 3.20 (3.20-3.26)
Collected reflections	
Total	410529
Unique	18507 (935)
Completeness (%)	99.9 (99.9)
*I*/*σ* (*I*)	14.3 (2.5)
*R*sym (%)	0.09 (0.7)
**Refinement statistics**	
*R* (%)	21.12
*R*_free_ (%)	28.73
∆bonds (Å)	0.01
∆angles (deg)	1.5
PDB ID	4KM3

#### Active site in the inactive conformation

Although the overall structure of *Sp*MetAP1a is similar to other MetAP structures in the catalytic domain, there is important difference near the active site. Not withstanding the confirmation of the extra loops (discussed below), a β-hairpin^198-211^ (equivalent region in *Ec*MetAP1a is 170-183) undergoes a conformational change and occupies the active site where the substrate methionine usually binds (Figure 3b). In the new conformation, the metal binding H199 moves away from the active site when compared to analogous residue (H171) in *Ec*MetAP1a. Due to this flip of the β-hairpin, residues in the loop region are forced to occupy the substrate-binding region (Figure 3b). Hence, substrate cannot bind to the enzyme in this conformation. To understand the flexibility of^98-211^β-hairpin in the crystal, intact crystals of *Sp*MetAP1a were subjected to the enzymatic activity. In the crystal form, the enzyme was inactive. However, crystals when crushed in the crystallization buffer, enzyme was active. This suggests, the overall structure and particularly the β-hairpin near the active site are not flexible in the crystal form. To demonstrate that in general, MetAP’s are active in the crystal form, *Ef*MetAP1a and *Mt*MetAP1c crystals were tested. Crystals of both these enzymes displayed activity (data not shown). Although these data suggest that the protein molecules in the crystals of the *Sp*MetAP1a are locked in the inactive conformation, further studies such as site directed mutagenesis and/or crystallization in a different crystal form would reveal the role of two inserts on the^98-211^β-hairpin and in turn on the activity of the enzyme.

#### ^103^Gly–Ser^129^ insert contains a Type 1 β-turn:

The insert region (^103^Gly–Gly^132^) forms the extension of the loop within a beta-hairpin (Figure 3a). Though less ordered and diffuse, clear electron density is visible for most of this insert in both the molecules^109^. Asp-Phe^117^ region forms a reverse β-turn. It is well characterized that asparagine residues in the β-turns are prone for glycosylation [14] confirming our prediction. The tyrosine kinase phosphorylation motif (^124^KKYT) is also well ordered and structurally placed closed to the glycosylation motif (Figure 3a). Apart from this insert, the smaller four amino acid insert (^63^AMMD) observed within the pita-bread fold extends the usually observed tight helical turn into a long loop (Figure 3c). Incidentally, the glycosylation motif, tyrosine phosphorylation motif and ^63^AMMD insert structurally fall in a straight line adjacent to the active site (Figure 3a).

#### Structural features of insert regions compared to other MetAPs

**Figure 4:**
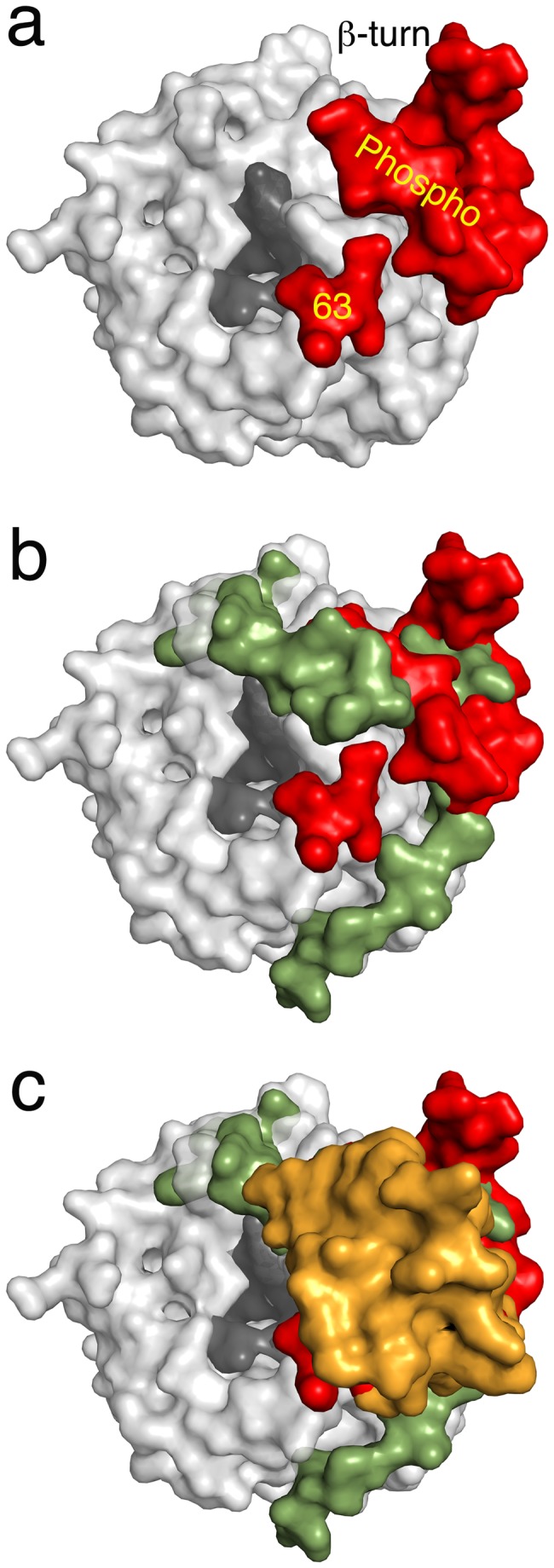
Genetic variations of MetAP’s with additional sequences. Catalytic domains are shown in light grey color while the entrance to the active site in the dark grey. a) Crystal structure of *Sp*MetAP1a. Two extra inserts are depicted in red. b) Overlay of *Sp*MetAP1a and *Mt*MetAP1c. Extension of the N-terminus of the *Mt*MetAP1c is shown in green. The inserts in the *Sp*MetAP1a and N-terminal extension in the *Mt*MetAP1c structurally align well suggesting of common function. c) Overlay of *Sp*MetAP1a, *Mt*MetAP1c and *Hs*MetAP2 structures. The insert domain of the later enzyme is shown in gold color. Note that all three extra modifications on the three different MetAP’s align at the same place reconfirming the common functional roles of these extra regions.

Structural alignment of *Sp*MetAP1a with Type Ib, Type Ic and Type II MetAPs provides interesting features. Both Type Ib and Type Ic MetAPs have N-terminal extensions but differentiated by the presence of zinc finger domain in the former (Figure 1a). Earlier, we have demonstrated that the common regions of N-terminal extensions between human MetAP1b (*Hs*MetAP1b) and *Mt*MetAP1c have similar structural features. Both these classes of proteins have conserved poly-proline motifs (P-X-X-P) that have tendency to participate in the protein-protein interactions with SH3 domain containing proteins (Figure 4b) [4,5]. The 60 amino acid insert region within the catalytic domain of Type II human MetAP (*Hs*MetAP2) forms a compact helical domain (Figure 4c). Surprisingly, inserts in the *Sp*MetAP1a, N-terminal extensions in the *Hs*MetAP1b, *Mt*MetAP1c and the insert in the *Hs*MetAP2 all share the same territory of the catalytic domain. Since some of these proteins, at least with eukaryotic origin are expected to localize at the ribosome exit-tunnel to co-translationally remove N-terminal methionine, the new insert regions observed in the streptococcal MetAP1a may also form some kind of protein-protein interactions.

## Discussion

Glycosylation is an important process in the biofilm formation during the infection of *Streptococcus pneumoniae* establishing the fact that post-translational glycosylation of proteins in this organism is critical [15]. In addition to glycosylation, tyrosine phosphorylation is also an essential post-translational modification for capsid formation of streptococci family [13]. The 27 amino acid insert in the streptococcal MetAP1a with possible glycosylation and phosphorylation modifications structurally aligns well with P-X-X-P motif region of Type Ib and Type Ic, suggesting important functions apart from removal of the initiator methionine. One possibility is that it could be useful in localization to a specific region in the cell for example to the cell membrane or to the ribosomes. In addition, it may also be involved in some signaling cascade. Though, it is not obvious from the sequence, due to the glycosylation or phosphorylation, if this protein is exported to the cell surface, the unique sequence of the extra-region of streptococcal MetAPs can be used as an antigen to develop vaccine against some of the deadly streptococcal strains.

**Figure 5:**
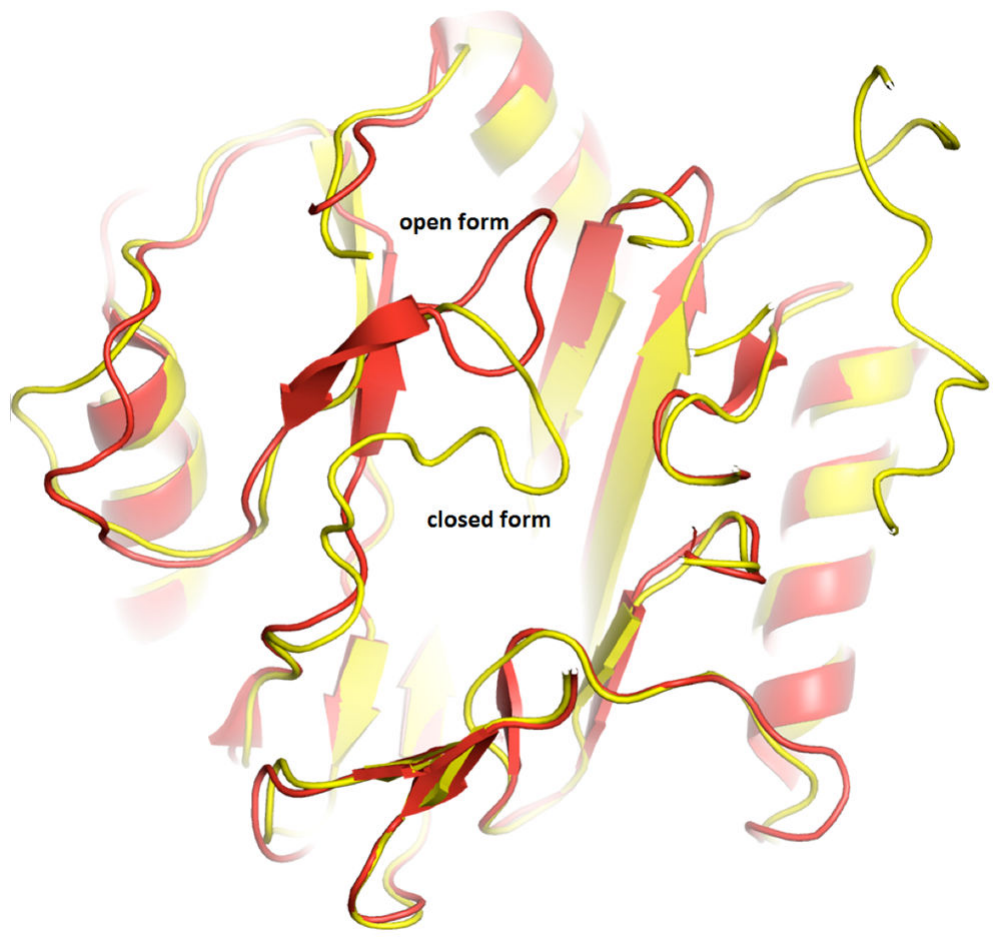
Inactive conformation of the *Sp*MetAP1a. Cartoon representation of the structural alignment of *Sp*MetAP1a (yellow) and *Ec*MetAP1a (red). Note that the β-hairpin loop collapses in to the active site there by the peptide with methionine on the amino terminus cannot bind to the enzyme.

This is the first crystal structure of MetAP determined in the closed/inactive conformation. However, the enzyme is active in solution showing strict specificity to methionine containing peptides. The flip of the beta-hairpin into the active site results in the occlusion of the methionine into the active site (Figure 5). This could be due to the crystal-packing artifact or it could be a representation of physiological switch between active and inactive forms induced by the two new insert regions, which are in close proximity to the active site (Figure 3). There are more than 55 structures of various MetAPs deposited in the protein databank (PDB) crystallized in different space groups and none of them displayed inactive/closed conformation. In addition, activity on the crystals point that these loops are not flexible in the crystal form.

## Conclusions

Here, we describe the crystal structure of a new genetic variant of the MetAP family of enzymes that are predominantly present in streptococcal bacteria. Important features of this variant are that it caries two inserts within in the catalytic region, one of which may undergo posttranslational modifications. The crystal structure of this enzyme also is unique since this is the first example of a MetAP crystallizing in the inactive form. Given differences with other bacterial and mammalian MetAPs, we believe that small molecules can target this genetic variant with specificity.

## Materials and Methods

### Cloning, expression and purification

*Sp*MetAP1a gene was amplified from the genomic DNA of *Streptococcus pneumoniae* (ATCC Catalog number: BAA-334d-5) using the following primer 5′-GGGGCTAGCATGATAACATTAAAATCAGCTCGTG-3′; 5′-GGGCTCGAGATAAGTTCCTTCTTCACCTTGGC -3′. The amplified product after digestion with NheI and XhoI was ligated into pET28a vector encoding N-terminal poly-His-tag. The construct was then transformed into *E. coli* BL21 (DE3) cells. Overnight 100 ml bacterial culture was inoculated to two liters of LB, which was then incubated at 37 °C at 250 rpm. At OD_600_ = 1.0, protein expression was induced with 1 mM isopropylthio-β-galactoside (IPTG) and further incubated for 16 h while shaking at 150 rpm at 25 °C. Cells were harvested by centrifuging at 6000 g and the cell pellet was stored at -80 °C until further use. Frozen 7.0 g cell pellet was re-suspended in 40 ml of +T/G buffer (50mM HEPES, pH 7.5, 0.5 M KCl, 10% glycerol, 0.1% Triton X-100, and 5 mM imidazole). All subsequent procedures were carried out at 4 °C. After complete re-suspension, cells were lysed by passing twice through a cell disruptor (LABMATE). Cell debris was cleared by centrifugation at 18,000 g for 30 min. The supernatant was loaded onto a Ni-NTA affinity column, which was pre-equilibrated with 200 ml of +T/G buffer. The column was further washed with 100 ml -T/G buffer (50 mM HEPES, pH 7.5, 0.5 M KCl, and 5 mM imidazole). Pure protein was eluted using -T/G buffer and 100 mM imidazole. The eluted protein was dialyzed into storage buffer (25 mM HEPES, pH 7.5, 5 mM methionine, and 150 mM KCl) followed by concentration to 15 mg/mL and stored at -80 °C. No attempts were made to cleave the His-tag or to purify further.

### Protein Crystallization and X-Ray Data Collection

The frozen protein was quickly thawed and diluted to 10 mg/ml in the storage buffer. Initial crystallization conditions were established from the Index Screen (Hampton Research, USA). Final crystals were grown at room temperature by using hanging drop vapor diffusion method using 1.4 M Na-K phosphate, pH 8.2. Diamond shaped crystals appeared in 7-10 days. Crystals were frozen in the presence of 20% glycerol in a nylon loop and mounted on the goniometer. Crystals diffracted the X-rays on a home source to a resolution of 3.2 Å. 180° data in 1° oscillation was collected and processed in *P*6_5_ space group [16] (Table 2). Structure was solved by molecular replacement using the coordinates from *E. feacalis* MetAP (*Ef*MetAP1a) (PDB ID: 3TB5) [17,18]. Refinement and modeling were performed using the REFMAC and coot [19,20]. Final graphical figures were generated using the Pymol [21].

### Biochemical characterization of *Sp*MetAP1a

#### Effect of different divalent metal ions on the enzyme activity

For studying the effect of different metal ions on the enzymatic activity, varying concentrations of divalent metal chlorides (1–1,000 µM) (Mg^2+^, Co^2+^, Zn^2+^, Mn^2+^, Ni^2+^) were added into the reaction mixture (25 mM Hepes, 150mM KCl, pH 7.5, 15 µM enzyme) and incubated at 30 °C for 30 minutes and then the substrate, Met-*p*NA was added to the reaction mixture. The production of *p*NA was monitored continuously by taking absorbance at 405 nm. The optimum concentrations of the best activating metal ions were selected for further characterizations.

#### Effect of pH

The optimum working pH of *Sp*MetAP1a was determined by screening buffers of different pH [Acetate buffer (pH 5.0, 5.5), Na-phosphate (pH 6.0, 6.5), MOPES pH 7.0, HEPES (pH 7.5, 8.0), Tris buffer pH 8.5, Na-Carbonate pH 9.0] at 25 mM concentration with 30µM of metal and 15µM of enzyme concentration. The activity was analyzed by the release of *p*NA from Met-*p*NA.

#### Determination of substrate specificity

Substrate specificity of *Sp*MetAP1a was determined by using para-nitroanilide (*p*NA) derivatives of different amino acids (Met-*p*NA, Gly-*p*NA, Arg-*p*NA, Pro-*p*NA, Leu-*p*NA, Val-*p*NA, Glu-*p*NA, Lys-*p*NA, Phe-*p*NA, Tyr-*p*NA and Ile-*p*NA). The assay was performed in 100 µL of 25 mM HEPES, 150 mM KCl, pH 7.5 in the presence of 30 µM of CoCl_2_ and 15 µM of enzyme concentration.

#### Enzyme kinetics

The Kinetic parameter of *Sp*MetAP1a was determined by using Met-*p*NA as a substrate in a 96-well clear polystyrene microplate. The 100 L reaction mixture contained 25 mM HEPES (pH 7.5), 150 mM KCl, 15 µM of enzyme, 30 µM of CoCl_2_ with different concentrations of Met-*p*NA (between 25 µM to 3.2 mM) with two fold increment. The reaction mixture was then incubated for 30 minutes at 30 °C. Km and Vmax were determined from slopes of various concentrations of the substrate by applying a non-linear curve fit. Kinetic analysis was performed using Sigma Plot (version 11.0) software. The turnover number and Kcat value were determined manually by applying the formula Kcat = Vmax/[ET], where Vmax is the maximum velocity and ET is the total enzyme concentration. The Data were fitted against the Michealis-Menten equation: V = Vmax × [S]/(Km + [S]), using Sigma Plot.

#### Measurement of protein activity with crystallization buffer

The assay was carried out in the crystallization buffer (1.4 M Na-K phosphate pH 8.2 and 25 mM HEPES, 150 mM KCl pH 7.5) with 5µM of protein and 10 µM of CoCl_2_. For enzyme activity, fluorogenic substrate methionine-7-amino-4-methylcoumarin (Met-AMC) (10 µM) was used because of its better sensitivity over Met-*p*NA. The reaction was carried out with excitation at 380nm and emission at 460 nm.

#### Measurement of activity with crystals

Crystals were fished out from the drop into 10 µl mother liquor (well solution). Since mother liquor is at equal or high concentration compared to the drop, crystals does not dissolve. After washing three to four times in the 10 µl of well solution, crystals were transferred to 100 µl of the mother liquor for the assay in the presence CoCl_2_ in a 96 well polystyrene black plate. Fluorogenic Met-AMC was added and the reaction was monitored. In a parallel reaction, after washing crystals, they were crushed and used for reaction as described above. The reaction was carried out at excitation wavelength of 380 nm and emission at 460 nm.
